# The Likelihood of Preventing Respiratory Exacerbations in Children and Adolescents with either Chronic Suppurative Lung Disease or Bronchiectasis

**DOI:** 10.3389/fped.2017.00058

**Published:** 2017-03-24

**Authors:** Kerry-Ann F O’Grady, Keith Grimwood

**Affiliations:** ^1^Institute of Health and Biomedical Innovation, Queensland University of Technology, South Brisbane, QLD, Australia; ^2^Menzies Health Research Institute Queensland, Griffith University, Gold Coast Health, Southport, QLD, Australia

**Keywords:** chronic suppurative lung disease, bronchiectasis, respiratory exacerbations, children, prevention

## Abstract

Chronic suppurative lung disease (CSLD) and bronchiectasis in children and adolescents are important causes of respiratory morbidity and reduced quality of life (QoL), also leading to subsequent premature death during adulthood. Acute respiratory exacerbations in pediatric CSLD and bronchiectasis are important markers of disease control clinically, given that they impact upon QoL and increase health-care-associated costs and can adversely affect future lung functioning. Preventing exacerbations in this population is, therefore, likely to have significant individual, familial, societal, and health-sector benefits. In this review, we focus on therapeutic interventions, such as drugs (antibiotics, mucolytics, hyperosmolar agents, bronchodilators, corticosteroids, non-steroidal anti-inflammatory agents), vaccines and physiotherapy, and care-planning, such as post-hospitalization management and health promotion strategies, including exercise, diet, and reducing exposure to environmental toxicants. The review identified a conspicuous lack of moderate or high-quality evidence for preventing respiratory exacerbations in children and adolescents with CSLD or bronchiectasis. Given the short- and long-term impact of exacerbations upon individuals, their families, and society as a whole, large studies addressing interventions at the primary and tertiary prevention phases are required. This research must include children and adolescents in both developing and developed countries and address long-term health outcomes.

## Introduction

Chronic (>4 weeks duration) wet cough in children implies increased airway secretions and lower airway infection (1). It is associated with often unrecognized morbidity and impaired quality of life (QoL) scores (2, 3). Within Australia, the most common cause of chronic wet cough in children is protracted bacterial bronchitis (PBB), followed by bronchiectasis (4). These two entities are at the opposite ends of a spectrum of overlapping conditions characterized by chronic wet cough, airway neutrophilia, and high bacterial densities in bronchoalveolar lavage (BAL) fluid cultures (5). They also share a common risk factor of impaired airway clearance facilitating microbial colonization of the lower airways (6), while their lower airway microbiota are dominated by strains of non-typeable *Haemophilus influenzae* (NTHi), *Streptococcus pneumoniae*, and *Moraxella catarrhalis* (1, 7–15).

Protracted bacterial bronchitis is defined as an illness with an isolated wet cough >4 weeks duration that resolves with a prolonged 2–4 weeks of appropriate oral antibiotic treatment, usually amoxicillin–clavulanate, and where other pointers suggestive of an alternative cause of cough are absent (16). It is a relatively newly defined diagnosis, but likely represents illnesses previously termed as chronic bronchitis, persistent endobronchial infection, or pre-bronchiectasis (16, 17).

By contrast, bronchiectasis has many underlying causes and is diagnosed when there is a chronic wet cough, which has a variable response to even prolonged courses of oral antibiotics, frequent respiratory exacerbations, and radiographic demonstration of irreversible dilatation of one or more bronchi (18). These children often have other accompanying respiratory symptoms and signs too, including growth failure, exertional dyspnea, digital clubbing, chest wall deformity, hyperinflation, and adventitial sounds on chest auscultation. The most common etiologies of bronchiectasis in children include post-infectious causes following recurrent and/or severe lower respiratory infections in early life, cystic fibrosis (CF), primary ciliary dyskinesia, immunodeficiency, retained foreign body, and chronic aspiration (12, 18).

Between the diagnostic spectrum of PBB and bronchiectasis are children with chronic suppurative lung disease (CSLD) who have the clinical symptoms and signs suggestive of bronchiectasis but lack the chest high-resolution computed tomography (cHRCT) scan evidence to support a diagnosis of bronchiectasis, such as an increased broncho-arterial ratio and absence of bronchial tapering while progressing toward the lung periphery (18, 19). Some of those categorized as having CSLD live in remote Indigenous communities in Australia and Alaska where undergoing cHRCT scans is difficult logistically and may be delayed until late in adolescence. Consequently, some of the younger children with CSLD may have undiagnosed bronchiectasis. In light of this and as management is the same for both diagnostic entities (19), this review focuses upon preventing respiratory exacerbations in children with either CSLD or bronchiectasis.

While published data are currently limited, recurrent episodes of PBB can also occur. In a study of 106 children with PBB followed for 2 years (20), 43.5% had >3 episodes per year and bronchiectasis was diagnosed subsequently in 8.1% of study participants (20). *H. influenzae* infection in the lower airways and recurrent episodes of PBB were independently associated with a future diagnosis of bronchiectasis [odds ratio (OR) 7.60, 95% confidence interval (CI) 1.53, 37.79 and OR 11.48, 95% CI 2.33, 56.50, respectively] (20). Nevertheless, children with recurrent PBB are not included in this review as the point where they progress to CSLD or bronchiectasis is yet to be determined, especially if they respond promptly to antibiotics and do not have additional symptoms or signs of respiratory disease. While CF is an important cause of chronic wet cough and bronchiectasis, children with CF are also excluded here given differences in the type of disease and concerns that the findings of studies in this patient population should not be extrapolated to those with bronchiectasis unrelated to CF (21).

## Acute Pulmonary Exacerbations

Acute pulmonary exacerbations are associated with disease severity (7), and although there are no direct data for CSLD or bronchiectasis in children, exacerbations are likely to contribute to a decline in lung function. Determinants of accelerated lung function decline in adults with bronchiectasis are frequency of hospitalized exacerbations, increased systemic inflammatory markers, and infection with *Pseudomonas aeruginosa* (22). Among other factors, increased mortality risk is associated with degree of lung function impairment (23). In children, studies from Australia and the United Kingdom reported that children with normal lung function at the time bronchiectasis was diagnosed maintained normal lung function 5 years later (24–26). Those children with poor lung function at diagnosis, although substantially improved, were still likely to have poor lung function 5 years afterward with the only significant predictor of pulmonary decline being the frequency of hospitalized exacerbations (25). With each hospitalized exacerbation, the forced expiratory volume in 1-s percentage (FEV_1_%) predicted decreased by 1.95% adjusted for time (25). Thus, interventions that minimize exacerbation frequencies are likely to be important in the overall management of bronchiectasis (19). In addition, preventing exacerbations will potentially reduce the associated economic and social costs. In the United States, between 1993 and 2006, the median cost for adult inpatient care was US$7,827 (27). Hospitalization rates increased significantly over the study time period with an annual percentage increase of 2.4% for males and 3.0% for females (27). At a New Zealand Hospital in 2004, half of the service’s pediatric bronchiectasis patients required at least one hospital admission per year (range 1–10) for exacerbations (28). The mean length of stay was 7 days (range 1–25). Eight percent of these children had regular three to four monthly admissions for 14 days to maintain respiratory status at a mean cost in 2004 of US$5,492 (not including theater time or costs for inserting peripherally inserted central catheter vascular lines) for each hospitalization (28).

## Definition

As with the paucity of data on CSLD and bronchiectasis in children in general (29), there is a dearth of literature defining an acute exacerbation. In adults, acute exacerbations of chronic obstructive pulmonary disease (COPD) are characterized by worsening dyspnea and increasing sputum volume and/or purulence (30). In adult bronchiectasis, exacerbations have similar features to that of COPD with increased cough frequency and sputum volume or purulence and are often associated with culturing respiratory bacterial pathogens in sputum (31). In research settings, particularly studies with exacerbations as the primary outcome, the definition may also include the need for hospitalization and intravenous antibiotics (32).

Clinical trials in children are currently using (33–36) similar definitions to that used in adults, including variations in symptoms of increased cough, dyspnea, increased sputum volume or color intensity, new chest examination or radiographic findings, deterioration in FEV_1_% predicted >10%, or hemoptysis. Not all definitions have, however, included the need for hospitalization or intravenous antibiotics.

In a study of 30 children with 115 exacerbations of bronchiectasis (25), increased cough frequency (88%) and change in cough character (67%) were the most frequent symptoms reported. Fever (28%), increased sputum volume (42%) and purulence (35%), and deteriorating chest auscultatory findings (58%) were also common, while changes in spirometry values compared to the stable state were not (25). Further work was undertaken from this cohort’s data (81 exacerbations included) (37) to develop a standardized definition of an exacerbation in children with bronchiectasis. Wet cough and cough severity (score ≥2) over 72 h were the best predictors of an exacerbation [receiver operating characteristic area under the curve of 0.85 (95% CI 0.79, 0.92) and 0.84 (95% CI 0.77, 0.91), respectively] (37). Sputum color, dyspnea, hemoptysis, chest signs, and chest pain were considered minor criteria and the addition of serum C-reactive protein (CRP), amyloid-A, and interleukin-6 improved the specificity and positive predictive value of the definition (37). The authors subsequently classified symptoms and laboratory measures into three sets of criteria (major, minor, and laboratory—Box 1) and then devised three options that could be used to define an exacerbation in bronchiectasis (Box 1). A limitation of the study is that there was a lack of an objective gold standard for diagnosing an exacerbation in bronchiectasis and hence the criteria were assessed against a pediatric pulmonologist’s definition that included change in symptoms and the need for additional treatment beyond the stable state. Further studies validating the definition in larger cohorts are now awaited.

Box 1 **Proposed criteria for defining a pulmonary exacerbation in children with bronchiectasis (37).**

(I) Major criteria
  ○ Significant frequency of cough (median cough score ≥2) over 72 h.
  ○ Wet cough for 72 h.
(II) Minor criteria
  ○ Sputum color ≥3 on BronkoTest™.
  ○ Parent/child perceived breathlessness.
  ○ Chest pain.
  ○ Auscultatory crackles.
  ○ Wheeze.
  ○ Hypoxia (oxygen saturation ≤93% by pulse oximetry).
(III) Laboratory criteria
  ○ CRP >3 mg/L on high sensitive testing.
  ○ Serum interleukin-6 >2 ng/L.
  ○ Serum amyloid-A >5 mg/L.
  ○ Raised peripheral blood neutrophil % (age appropriate).

The three combinations considered the best to define an exacerbation:

- (Option-A) *One major* PLUS any *one laboratory criteria* positive [sensitivity 63%, specificity 94%, AUC 0.784, *p* < 0.001; positive predictive value (PPV) 91%, negative predictive value (NPV) 71.6%], OR
- (Option-B) *Two major* criteria positive (sensitivity 92.6%, specificity 75.3%, AUC 0.84, *p* < 0.001; PPV 79%, NPV 91%), OR
- (Option-C) *One major* PLUS *any two minor* criteria positive (sensitivity 95%, specificity 75%, AUC 0.84, *p* < 0.001; PPV 78.5%, NPV 93.75%).

## Etiology

The exact cause of exacerbations in young children with CSLD or bronchiectasis is not well understood, and it is unclear whether these are new infections, a resurgence of chronic infection, or a combination of both (31, 38). Part of the problem is that despite their regular use in clinical practice, upper airway secretions collected by throat or cough swabs do not reliably predict organisms within the lower airways (9), especially as the potential pathogens of interest (NTHi, *S. pneumoniae*, and *M. catarrhalis*) are also found commonly in the upper airway spaces of healthy children (39). Repeated bronchoscopies with multi-lobar BAL to collect lower airway specimens before, during, and following acute exacerbations in children too young to reliably expectorate sputum would be ideal, but impractical given their invasive nature, including the need for repeated sedation or general anesthesia. Hence the data required to confidently assign causality in research and patient care are limited.

*Haemophilus influenzae, S. pneumoniae*, and *M. catarrhalis* are frequently isolated at high densities (≥10^4^ colony-forming units/mL) from BAL specimens collected from children and adults during an acute exacerbation, but mixed infections are also common (9, 13), and few studies have searched systematically for viruses. *P. aeruginosa* and non-tuberculous mycobacteria are uncommon in children with bronchiectasis and, when present, raises the possibility of undiagnosed CF while also being associated with more severe underlying bronchiectasis and co-morbidities (13). A small study of 69 children with bronchiectasis that included 900 child-months of follow-up (40) identified at least one respiratory virus in nasopharyngeal aspirates in 48% of exacerbations, most commonly human rhinovirus (54% of virus-positive events). Children with virus-positive exacerbations were more likely to require hospitalization (59 vs 32.5%; *p* = 0.02), have fever (OR 3.1, 95% CI 1.2, 11.1), hypoxia (OR 25.5, 95% CI 2.0, 322.6), chest signs (OR 3.3, 95% CI 1.1, 10.2), and raised CRP (OR 4.7, 95% CI 1.7, 13.1), when compared with virus-negative exacerbations (40).

In a cross-sectional study of 245 children diagnosed with PBB or mild bronchiectasis undergoing bronchoscopy and BAL for clinical indication (median age 30 months) (41), a standard respiratory panel for viruses using polymerase chain reaction assays was performed on all specimens. Human adenovirus (HAdV) was the most common virus detected, being identified in 40 children; influenza virus was detected in 3 children, parainfluenza virus in 12, respiratory syncytial virus in 11, and human metapneumovirus in 5 children (41). HAdV detection was more common in the young age groups (*p* = 0.001) and was positively associated with each of the three major bacterial pathogens (41). However, this association disappeared after adjustment for age and, given both the cross-sectional nature of the study with a lack of controls, a causal association between detecting these viruses and clinical illness at the time of bronchoscopy cannot be determined.

Similarly, there are very few studies examining the risk factors for exacerbations of CSLD or bronchiectasis. A study of 93 Indigenous children from Alaska and Australia with CSLD or bronchiectasis (42) reported 74% of children experienced >2 exacerbations over a 3-year period. In this study, the factors associated with recurrent episodes were young age (<3 years), hospitalization for an acute exacerbation in the first-year of life, and pneumonia or hospitalization for an acute exacerbation in the year preceding enrollment (42). Exacerbations are also more frequent in severe bronchiectasis, and one study of 111 children reported that intensive medical intervention reduced the annual exacerbation rate by 56%; however, children still experienced a mean of 2.9 episodes per year (43).

## Prevention

Preventing exacerbations and reducing severity, are important goals in managing children with CSLD or bronchiectasis in order to maintain lung health and enhance their QoL (19). The cornerstone of CSLD management is a combination of airway clearance techniques and antibiotic therapy, with or without other therapies such as anti-inflammatory agents and bronchodilators (31, 38, 44). However, moderate or high-quality studies in children for either CSLD or bronchiectasis are limited (45), including those addressing broader approaches such as vaccines, health promotion, and chronic disease management strategies.

### Pharmacological Agents

#### Antibiotics

A Cochrane review of antibiotic efficacy for preventing recurrent lower respiratory tract infections in high-risk children aged <12 years (46) identified a lack of evidence supporting prophylactic antibiotics and emphasized the need for high-quality trials to be conducted. That review included three studies involving children infected with human immunodeficiency virus, four with CF, and one each with children with sickle cell disease, cancer, and low birth weight neonates in a pediatric intensive care unit with underlying respiratory disorders (46). The only studies that addressed acute respiratory exacerbations were three of the CF studies; one (82 children) used ciprofloxacin in conjunction with colistin (polymyxin E) (47) and two (total of 312 children) used azithromycin (48, 49). The review excluded five studies [four in children with CF (50–53) and one involving children with either CSLD or bronchiectasis (54)] on the grounds that the children were already infected (51–54) or where infection could not be discounted (50). Given the underlying nature of these endobronchial disorders, where infection has a central pathogenic role, the justification for excluding the five studies is unconvincing, while the basis for being currently infected by unidentified organisms is also unclear.

In the Australian study excluded from the review (54), 89 children with either CSLD or bronchiectasis were randomized to receive either azithromycin (30 mg/kg once-a-week) or placebo for up to 24 months. Children receiving azithromycin had significantly lower exacerbation rates (incidence rate ratio 0.50; 95% CI 0.35, 0.71) than the control group (54). However, children in the azithromycin group also developed significantly higher carriage of azithromycin-resistant bacteria (19 of 41, 46%) than those receiving placebo (4 of 37, 11%; *p* = 0.002) (54). Acquiring macrolide-resistant organisms was more prevalent in those who were poorly adherent (76%) than those complying with their therapy (52%; OR 2.94, 95% CI 1.23, 7.14) and post-intervention *Staphylococcus aureus* strains remained resistant to macrolides (55). These findings support a role for directly observed therapy in individuals and communities where adherence may be suboptimal, which will then help to ensure the benefits of long-term antibiotics, while minimizing potential harm from acquiring antibiotic-resistant organisms. A dose–response relationship between azithromycin use and an increase in macrolide-resistant strains of *S. pneumoniae* and *S. aureus* in the nasopharynx was also observed in a prospective study of 79 remote Australian children with CSLD or bronchiectasis conducted between 2004 and 2008 (56). These findings are consistent with those of the 2015 Cochrane review (57), which reported a threefold increase in antibiotic-resistant bacteria.

The 2015 Cochrane review of the efficacy of long-term antibiotics (≥4 weeks duration) in children and adults with bronchiectasis (57), identified only three studies (33, 58, 59) that included children (total 148 participants), and one did not report exacerbations (58). Of the remaining two, one used oral azithromycin and is described above (54), and the second used either oral roxithromycin or placebo in 25 children for 12 weeks (59). This latter study failed to demonstrate a statistically significant difference in exacerbation rates, likely a result of the small sample size and short duration of follow-up, although the point estimate of effect provided a weak signal toward a reduction (OR 0.16, 95% CI 0.01, 3.60) (59). The study did, however, report significantly lower sputum purulence scores in the antibiotic group compared with those receiving placebo (1.39 ± 0.6 vs 2.17 ± 0.72; *p* < 0.01) (59). With respect to short-term antibiotics (<4 weeks duration), a further Cochrane review (60) identified no studies in children and, therefore, concluded there was no evidence to support this approach for reducing the severity and frequency of exacerbations in children with bronchiectasis.

#### Expectorants, Mucolytics, and Mucokinetics

Retained mucus in the lower airways promotes bacterial growth, persistent airway inflammation, and bronchial wall injury (61). Hence reducing mucus retention is an important aspect of airway clearance techniques. Mucoactive agents include expectorants, mucolytics, and mucokinetic agents.

Expectorants aim to increase the volume of airway water or secretion in order to increase the effectiveness of cough (62) and include both over-the-counter (OTC) cough medications and inhaled hyperosmolar saline and mannitol. There is no good evidence for the use of OTC cough medications and expectorants in children (63), and concerns about adverse events, including some reports of infant deaths (64), led to several countries recommending OTC cough medications should not be given to young children. Inhaled hyperosmolar agents, including hyperosmolar saline and mannitol, potentially alter the physical properties of mucus by increasing water in the airway lumen and disrupting mucin networks (65). While there is evidence to support the role of hyperosmolar agents in children with CF (62), there are insufficient data to support their use in children with CSLD or bronchiectasis (45).

Mucolytics make mucus less viscous so that it can be more easily expectorated, and they are available in both oral and inhaled forms. This group of drugs includes *N*-acetylcysteine, ambroxol (3), sobrerol, carbocysteine, sobrerol, letosteine, cithiolone, iodinated glycerol, *N*-isobutyrylcysteine, myrtol and erdosteine, and recombinant human DNase (RhDNase) (62). The exact mechanism of action of most mucolytics is unclear, except for RhDNase that targets neutrophils at the site of infection in the lungs (66, 67). There have been no pediatric trials of mucolytics in children with CSLD or bronchiectasis (66). Moreover, RhDNase is not recommended in either children or adults with bronchiectasis given a study in adults reporting its negative effects on lung function and exacerbation rates (68).

Mucokinetics increase the effectiveness of cough, either by increasing expiratory cough airflow or by removing secretions from the airway walls; aerosol surfactant is one of this class of medications (62). However, as with the other pharmacologic airway clearance therapies described above, there is no high-level evidence to support their use in children with CSLD or bronchiectasis (69).

#### Bronchodilators

Bronchiectasis may be associated with an obstructive ventilatory defect, which may worsen during an exacerbation (70). Although patients with bronchiectasis may also have asthma (71) and the presence of bronchiectasis may worsen asthma exacerbations (72), small adult studies suggest the airflow limitation is not readily reversible (73). There are, however, scarce data on the prevalence of asthma in children with CSLD or bronchiectasis, although there are suggestions that children with either CSLD or bronchiectasis have been misdiagnosed with asthma previously (19). There are no trials of bronchodilators in children with CSLD or bronchiectasis in the absence of a confirmed diagnosis of asthma, and their use in this group of children is not recommended currently (74).

#### Corticosteroids

Airway inflammation is a key characteristic of both CSLD and bronchiectasis where the resultant symptoms can be similar or falsely attributed to asthma (75). While the data are limited, the presence of asthma-like symptoms in the presence of bronchiectasis has been associated with accelerated lung function decline (76–78). Inhaled corticosteroids are commonly used to control asthma symptoms, including preventing exacerbations. This class of drugs demonstrates broad anti-inflammatory actions, which can occur either rapidly or over a period of hours or days (79). Side effects are, however, not uncommon, and there are data to suggest children with persistent asthma receiving daily high-dose inhaled corticosteroids have an increased risk of linear growth faltering over a 12-month treatment period (80). Furthermore, for this strategy to be effective treatment, adherence is especially important (81) and, while data are limited in children with CSLD or bronchiectasis, non-adherence to inhaled corticosteroids regimens in adults with bronchiectasis has been documented (81, 82), and is known to be problematic in children with asthma, particularly adolescents. A Cochrane review of inhaled corticosteroids in children and adults with bronchiectasis identified no pediatric studies, and hence there is no available evidence to support their use (75). However, a small study of inhaled corticosteroid withdrawal over a 12-week period in 27 children with bronchiectasis (83) reported a significant increase in bronchial hyper-reactivity after ceasing the drug (37.0 vs 46.8%, *p* = 0.016) and a decrease in neutrophilic apoptosis in induced sputum (42.8 vs 20.2%, *p* = 0.03), but no change in sputum inflammatory markers or in the number of infectious exacerbations (83). Larger studies are required to confirm these findings and to determine whether they are of any clinical significance. By contrast, the role of systemic corticosteroids in children or adults with CSLD or bronchiectasis has not been examined (84, 85).

#### Non-Steroidal Anti-inflammatory Agents

Non-steroidal anti-inflammatory drugs (NSAIDs) are a class of medications that act as non-selective inhibitors of the enzyme cyclo-oxygenase and induce analgesic, antipyretic, and anti-inflammatory effects. Both oral and inhaled NSAIDs are available. A recently updated Cochrane review of inhaled NSAIDS in children and adults with bronchiectasis identified no relevant trials of NSAID efficacy in children and concluded there was no evidence to either support or refute their use in bronchiectasis (86). Another review of the efficacy of oral NSAIDs published in 2007 identified no trials in children or adults and could, therefore, make no recommendations regarding their use (87).

#### Vaccines

With respect to pathogens associated with respiratory illnesses, pediatric vaccines currently exist for influenza, *S. pneumoniae* (23 valent polysaccharide vaccine and 7, 10, and 13 valent pneumococcal conjugate vaccines), *Bordetella pertussis, H. influenzae* type b, measles, and varicella. The 10-valent pneumococcal vaccine uses Protein D, an outer membrane protein derivative from NTHi strains, as the conjugate. There is currently no evidence for any of these vaccines in preventing acute exacerbations of either CSLD or bronchiectasis in children (88), primarily because the trials have not been done. A randomized controlled trial (RCT) of the 10-valent pneumococcal Protein D conjugate vaccine in children with CSLD or bronchiectasis using acute exacerbations as the primary endpoint has concluded recently in Australia (36), although the results were not yet published at the time of this review. The need for a safe and effective vaccine targeting NTHi for children is being recognized increasingly as a priority preventive and therapeutic intervention (89–91). Despite the lack of evidence for preventing exacerbations, children with chronic lung diseases are at high risk for severe infections from most of these organisms (92–94), and disease management should include ensuring children receive on-time and age-appropriate immunizations in all countries where these vaccines are recommended through national immunization programs.

### Non-Pharmacological Airway Clearance Techniques

Non-pharmacological airway clearance techniques are strategies that aim to clear the airway of secretions, improve gas exchange, and hence reduce the risk of infection in the lower airways (95). The core components involve postural drainage, percussion, vibration of the chest wall, and coughing (96), and specific techniques include active cycle of breathing techniques, forced expiration techniques, autogenic drainage, postural drainage, oscillating positive expiratory pressure, high frequency chest wall oscillation, and exercise and/or pulmonary rehabilitation (96). While a Cochrane review found these techniques have clinically important effects on health-related QoL scores in adults (97), it identified only one study in children with bronchiectasis (98). This study involved only nine children with bronchiectasis and the intervention was oscillatory positive expiratory pressure administered three times a day with lung function measured after a 3-month period. The study did not include exacerbations as an outcome. Despite the lack of trials, non-pharmacological airway clearance techniques are recommended in managing children with CSLD or bronchiectasis (71, 74, 99) given the need to reduce mucus retention in the lower airways. The efficacy of these treatments with respect to preventing exacerbations is unknown.

### Chronic Disease Management Plans

The complexity of managing patients with chronic diseases necessitates a multi-disciplinary approach that involves shared decision-making and includes the patient and their carers/family in developing treatment plans (100, 101). Personalized chronic disease management plans involve joint goal setting and agreement on actions necessary to achieve those goals (102). A systematic review of 19 studies involving 10,856 adults with a range of chronic health conditions concluded that modest improvements can be made through personalized management plans in physical and psychological parameters, as well as in patient capacity to self-manage their conditions (102). There appears to be no published studies of the efficacy of chronic disease management plans in preventing exacerbations of CSLD or bronchiectasis in children. A systematic review of action plans for adults with COPD that included limited self-management education identified improvements in the recognition of, and initiation of treatment for, acute exacerbations, but there was no evidence for reduced health-care utilization or improved health-related QoL (103). A review of shared care of people with chronic diseases between primary and specialty health services concluded there was insufficient evidence for the benefits of that approach other than improved prescribing (104).

### Health Promotion Strategies

Optimizing healthy growth and development is important for all children; however, additional efforts are required for children with CSLD or bronchiectasis given the links between respiratory infections and key factors, such as nutrition, exposure to environmental toxicants (particularly tobacco smoke), and healthy weight.

#### Optimize Nutritional Status

Nutritional deficiencies in children may lead to poor lung function, increased susceptibility to infections, and a greater likelihood of acute illnesses in childhood and chronic illness in adulthood (105). Micronutrient deficiencies, particularly of vitamins A, C, and D, folic acid, and the trace elements, zinc and iron, have been linked to many childhood infectious diseases, including respiratory infections (105). There is increasing interest in the role of vitamin D in respiratory diseases given its modulatory and regulatory role in inflammation and immunity (106). There are some data suggesting adults with chronic lung diseases are frequently deficient (107) and that deficiency is associated with disease severity (108). However, the studies supporting a role for vitamin D in protection against acute and chronic respiratory infections are associative and come from observational studies. A recent systematic review and meta-analysis of seven RCT of vitamin D supplementation in 6,503 children aged <18 years found no evidence to support their use for preventing or treating acute respiratory infections in healthy children (109). Interestingly, in this meta-analysis, vitamin D supplementation reduced the risk of asthma exacerbations by 74% [relative risk (RR) 0.26, 95% CI 0.11, 0.59], although this result needs to be interpreted cautiously as it involved two small studies of 430 and 48 subjects, respectively, and where significant heterogeneity was observed. Thus larger RCT are needed to determine whether vitamin D supplementation can reduce the risk of acute asthma exacerbations and if it should also be studied in children with other chronic lung diseases, including CSLD and bronchiectasis.

In addition, high intake of fructose-containing beverages such as soft drinks and fruit juice has been associated with increased prevalence of asthma in children (110, 111) and with chronic bronchitis in adults (112). Currently, there are no data on the role of micronutrient deficiencies, micronutrient supplementation, and high fructose intake in children with either CSLD or bronchiectasis. However, promoting a healthy diet is important for all children. A study of 141 Polish children with recurrent bronchitis found higher levels of body fat and muscle mass deficiency compared to reference ranges for healthy children (113). Whether this result reflects cause or effect is unknown, but while there are no efficacy trials of weight reduction in preventing exacerbations of pediatric CSLD or bronchiectasis, there are some data, albeit limited, to suggest an effect in adults with asthma (114). Given the global rise of high calorie diets and associated obesity, and the complex socioeconomic circumstances where that has occurred, reducing calorie intake in children requires a multi-sectorial, population-based approach that includes strong government policy (115).

#### Reduce Exposure to Tobacco Smoke

Active and passive exposure to tobacco smoke is well-known as important risk factors for respiratory diseases. Children of parents who smoke are at approximately twice the risk of being hospitalized for respiratory illnesses (116). Hence avoiding exposure is not only beneficial to lung health but reduces the risk of the multitude of other adverse health outcomes associated with smoking. While knowledge around the effects of a child’s passive exposure to tobacco smoke is improving in some settings (117), challenges remain in improving community and smoker perceptions of the risk to children, including among families of children with chronic lung diseases (117–119). Several strategies have been studied to reduce household tobacco smoke exposure in children, including motivational interviewing (with or without education) (120), maternal and child health nurse interventions (121), and family-centered interventions (122); however, the effectiveness of many strategies is modest at best (123).

Efforts are required to ensure children with CSLD or bronchiectasis do not take up smoking, particularly as they enter the vulnerable adolescent and young adult years (124). This must include efforts to prevent the use of e-cigarettes, which are becoming increasingly popular among adolescents despite emerging evidence to suggest adverse health effects (125). In a study of 2,086 North American adolescents of e-cigarette use and reported chronic cough, sputum production, or bronchitis symptoms in the previous 12 months (126), the risk of symptoms was almost twofold higher among past users (OR 1.85, 95% CI 1.37, 2.49), compared to never users, and by 2.02-fold (95% CI 1.42, 2.88) among current users. The risk also increased with frequency of current use (126).

#### Physical Activity

Promoting physical activity in children and adolescents should be standard practice for health-care providers given its well-known benefits to overall health and well-being. However, chronic illness can impact on activity levels in children, potentially affecting disease progression (127, 128). People with advanced lung disease experience static and dynamic hyperinflation due to airflow limitation, leading to reduced tidal volumes, increased dead space ventilation, and exertional dyspnea making exercise difficult (127). Physical activity provides higher health-related QoL scores (129) and greater aerobic capacity (130).

A study of an 8-week exercise training program in 55 adults with bronchiectasis who were subsequently followed for 12 months (131) reported fewer exacerbations in the intervention group compared to controls (RR 0.69) (95% CI 0.49, 0.98) as well as fewer exacerbations that required antibiotics. There are no data on the patterns of physical activity in children with CSLD or bronchiectasis, nor are there data on the effectiveness of physical activities in reducing exacerbations in these children.

## Conclusion

Chronic suppurative lung disease and bronchiectasis in children and adolescents are serious illnesses with significant adverse impacts on the child, their family, the health-care sector, and broader community. Despite this, there has been scant attention paid to the spectrum of disease, its management, and prevention in comparison to CF and chronic lung diseases in adults. Exacerbations of CSLD and bronchiectasis in children are important predictors of lung function decline and are associated with significant morbidity. Preventing exacerbations should, therefore, be the cornerstone of long-term disease management. However, there are only limited data on preventing acute respiratory exacerbations in children with CSLD or bronchiectasis. Hence management is currently informed largely by clinical opinion and extrapolating data from adults and/or children with CF and asthma, Unfortunately, such an approach is not without risk, where treatments highly successful in CF patients such as inhaled rhDNase, tobramycin, aztreonam, and colistin have provided disappointing results in adults with bronchiectasis (132). A standardized, validated definition of an acute exacerbation in children with CSLD and bronchiectasis is needed urgently as an outcome measure in clinical trials and to help guide clinical practice. In a similar vein, studies to determine when recurrent episodes of PBB should be classified and managed as CSLD are also required. Substantially more, and larger studies, are needed to determine the efficacy and effectiveness of both pharmacological and non-pharmacological interventions to prevent exacerbations. These studies must also include health-related QoL assessments and cost-effectiveness of the interventions. Given the burden of disease is likely to be highest in low- and middle-income countries, measures to prevent exacerbations must be translated to low socioeconomic and disadvantaged community and health-care settings that do not have access to the drugs and technology available in high-income nations.

For the clinician, the lack of robust well-designed, intervention-based RCT to guide prevention of acute exacerbations of CSLD and bronchiectasis in children and adolescents means that guidelines are more consensus than evidence-based. We follow the Thoracic Society of Australia and New Zealand (TSANZ) guidelines (19, 74) where children and adolescents are seen three times monthly and where we recommend they practice regular airway clearance techniques in consultation with a pediatric respiratory physiotherapist, encourage physical activity, monitor growth and optimize nutrition, counsel against active and passive tobacco smoke exposure, promote avoidance of other environmental inhaled toxicants, and ensure they receive their vaccinations according to the National Immunization Program, including the pneumococcal and annual seasonal influenza vaccines. At the same time, co-morbidities, such as underlying immunodeficiency or chronic aspiration, are often managed with the aid of other specialty services. Children and adolescents who are frequent exacerbators (≥3 exacerbations and/or ≥2 respiratory hospitalizations in the previous 12 months) are prescribed low-dose oral azithromycin for a trial period of 12–24 months according to the TSANZ guidelines. Selected patients are offered inhaled hypertonic saline as a mucoactive agent and/or an inhaled antibiotic, usually tobramycin, if exacerbations continue, especially if they have a chronic *P. aeruginosa* infection of their lower airways. These agents are trialed separately and if tolerated are continued long term. Finally, bronchodilators and inhaled corticosteroids are administered only to those with an established diagnosis of asthma and are not used routinely for other patients with CSLD or bronchiectasis.

## Author Contributions

KO devised the scope of the manuscript, performed the literature reviews, and wrote the first and final draft. KG made a substantial contribution to the content and writing of the manuscript. Both the authors approved the final version.

## Conflict of Interest Statement

The authors declare that the research was conducted in the absence of any commercial or financial relationships that could be construed as a potential conflict of interest.

## Abbreviations

AUC, area under the curve; CF, cystic fibrosis; cHRCT, chest high-resolution computed tomography; CI, confidence interval; COPD, chronic obstructive pulmonary disease; CRP, C-reactive protein; CSLD, chronic suppurative lung disease; FEV_1_%, forced expiratory volume in one-second percentage; NPV, negative predictive value; NSAIDs, non-steroidal anti-inflammatory drugs; OR, odds ratio; OTC, over-the-counter; PBB, protracted bacterial bronchitis; PPV, positive predictive value; QoL, quality of life; RCT, randomized controlled trial; rhDNase, recombinant human DNase; RR, relative risk; TSANZ, Thoracic Society of Australia and New Zealand; US, United States.
